# What Aspects of Illness Influence Public Preferences for Healthcare Priority Setting? A Discrete Choice Experiment in the UK

**DOI:** 10.1007/s40273-021-01067-w

**Published:** 2021-08-19

**Authors:** Liz Morrell, James Buchanan, Sian Rees, Richard W. Barker, Sarah Wordsworth

**Affiliations:** 1 Health Economics Research Centre, Nuffield Department of Population Health, University of Oxford, Old Road Campus, Roosevelt Drive, Oxford, OX3 7LF UK; 2 Oxford-UCL Centre for the Advancement of Sustainable Medical Innovation, Radcliffe Department of Medicine, University of Oxford, Oxford, UK; 3 NIHR Oxford Biomedical Research Centre, University of Oxford, Oxford, UK; 4 Oxford Academic Health Science Network, Oxford, UK

## Abstract

**Background:**

Decisions on funding new healthcare technologies assume that all health improvements are valued equally. However, public reaction to health technology assessment (HTA) decisions suggests there are health attributes that matter deeply to them but are not currently accounted for in the assessment process. We aimed to determine the relative importance of attributes of illness that influence the value placed on alleviating that illness.

**Method:**

We conducted a discrete choice experiment survey that presented general public respondents with 15 funding decisions between hypothetical health conditions. The conditions were defined by five attributes that characterise serious illnesses, plus the health gain from treatment. Respondent preferences were modelled using conditional logistic regression and latent class analysis.

**Results:**

905 members of the UK public completed the survey in November 2017. Respondents generally preferred to provide treatments for conditions with ‘better’ characteristics. The exception was treatment availability, where respondents preferred to provide treatments for conditions where there is no current treatment, and were prepared to accept lower overall health gain to do so. A subgroup of respondents preferred to prioritise ‘worse’ health states.

**Conclusion:**

This study suggests a preference among the UK public for treating an unmet need; however, it does not suggest a preference for prioritising other distressing aspects of health conditions, such as limited life expectancy, or where patients are reliant on care. Our results are not consistent with the features currently prioritised in UK HTA processes, and the preference heterogeneity we identify presents a major challenge for developing broadly acceptable policy.

**Supplementary Information:**

The online version contains supplementary material available at 10.1007/s40273-021-01067-w.

## Key Points for Decision Makers

**Table:** 

There are attributes of illness that members of the public identify as distressing, and lead to strong public reaction when new technologies for such conditions are not funded following technology appraisal. We performed a choice study to determine the influence of such attributes on the value placed on alleviating illness.
Our results suggest a preference among the UK public for prioritising conditions where there are no other treatments available; however, we do not find an overall preference for prioritising other distressing aspects of ill-health such as shortened life expectancy or reliance on care.
The findings from this study do not align with the characteristics given extra weighting in current UK policy, and this mismatch should be examined further.

## Introduction

Health care decision makers have to make difficult choices about how to allocate health system budgets. In countries with formal health technology assessment (HTA) processes, funding decisions for new technologies are based partly on assessing their cost effectiveness versus comparator interventions. In the UK, there is a fixed healthcare budget, and cost-effectiveness is considered from the perspective of the health service. Health gain is measured using a common metric, the quality-adjusted life-year (QALY), and the cost per QALY is compared with a threshold representing the health gain from interventions that would be displaced [1]. These analyses assume that the goal of the health system is to maximise population health and that a given health gain is equivalent, regardless of who benefits (the ‘QALY = QALY’ assumption) [2]. However, the agencies responsible for these decisions (such as the National Institute for Health and Care Excellence [NICE] in England) do not solely consider evidence on cost effectiveness, but may allow for additional factors, such as innovation, or reducing health inequalities [3–5].

Such factors are typically considered through deliberation, but some are formalised in policy, often in response to public debate [6]. For example, in England, treatments for patients with short life expectancy are prioritised by giving additional weight to QALYs gained (NICE’s ‘end of life’ criteria [7]), following media focus and strong public reaction to decisions not to fund new cancer drugs [8–11]. Similarly, the Scottish Medicines Consortium (SMC) has a specific appraisal route for treatments for end of life or rare conditions, to identify elements of value that would otherwise not be accounted for in the standard cost-per-QALY framework [12]. Other countries have made similar adjustments [13].

The Value-Based Pricing initiative in England (2010–2014) aimed to broaden the range of factors considered in NICE’s appraisals and was expected to lead to more drugs being considered as cost effective [14]. The initiative proposed severity of illness, wider social benefits, and incentivising innovation as factors for inclusion [15]. Attempts to operationalise these factors failed to find broad stakeholder agreement and the proposals were not implemented. Nevertheless, there remain concerns regarding the breadth of outcomes considered in HTA appraisals. Indeed, NICE initiated a review of its methods of technology appraisal in 2019. Topics approved by NICE’s Board for consideration in the review include the methods used to measure quality of life, the factors used in decision making in addition to clinical and cost effectiveness, and how to consider a wider range of sources of evidence [16].

The empirical literature on factors that could be used to ‘weight’ QALY gains includes studies exploring characteristics of the patient, the intervention, or the illness. Patient characteristics include age, lifestyle, disadvantage and prior care [17–22]. Features of the intervention have included the type of health gain, certainty and size of benefit, and innovation [17, 20–25]. Studies evaluating characteristics of illness have largely focused on measurable attributes with policy relevance, such as disease rarity, life expectancy, severity of illness and quality of life [19, 21–23, 26, 27]. However, there is little information on the factors that members of the public would give precedence to—based on their own experience—in decisions on priority for funding.

This paper reports the results of a study aiming to address this question, using a discrete choice experiment (DCE). The study aimed to determine the relative importance of attributes of illness that influence the value placed on alleviating that illness. Specifically, we hypothesise that the public may place a higher value on alleviating illness with particularly distressing characteristics, such as limited life expectancy. A DCE is an appropriate method because it presents choices as ‘bundles’ of multiple characteristics, reflecting the complexity of healthcare choices. By asking respondents (here, members of the public) to make choices between alternatives described by a set of attributes, a DCE provides quantitative information on the relative importance of these attributes and the trade-offs between them.

## Methods

Our study design and data collection and analysis followed the checklist developed by the International Society for Pharmacoeconomics and Outcomes Research (ISPOR) Good Research Practices for Conjoint Analysis Task Force [28], which includes DCEs.

### Research Question

Our aim was to understand the relative importance of attributes of illnesses in contributing to the value that the public places on alleviating that illness.

### Defining Attributes and Levels

The alternatives presented to respondents were described by six attributes, with the aim of capturing a sufficient range of features of illness without making the choices excessively complex for respondents. Similar numbers of attributes have been found to be acceptable to respondents in other studies related to priority setting in the UK [17, 23, 26, 27] and elsewhere [19, 21, 22, 25].

We aimed to identify attributes of illness that the public find particularly distressing. Although attributes of public experience of cancer are well-described in the literature (reviewed by Vrinten et al. [29]) we found little evidence for other conditions. We therefore undertook qualitative research to understand the features that shape how serious illnesses (such as heart disease, dementia and infectious diseases) are perceived by the public (reported separately [30]). As a second source, and given our ultimate interest in QALY weighting, we sought to identify aspects of illness that are perceived to be inadequately captured by HTA processes in the UK. We used the SMC’s ‘Patient and Clinician Engagement’ process as a case study, as the process aims specifically to elicit such features (also reported separately [31]). Third, we used a systematic review on public views on weighting factors for priority setting, by Gu et al. [32]. Features generated from these sources were compared and aligned (by LM and JB) to reflect common ideas. We selected features that occurred in multiple sources and these were refined into attributes through discussion among the authors. Full details are provided in the electronic supplementary material (ESM). The attributes were reviewed by a group of representatives from patient advocacy charities who were familiar with a range of serious illnesses (e.g., cancer, dementia and musculoskeletal diseases) to confirm that these attributes covered a sufficient range of features and were described clearly.

The selected attributes and levels, and the rationale for their inclusion, are shown in Table 1. The attribute levels are ordered from ‘worst’ to ‘best’ in terms of public experience and this terminology is used throughout the paper. All variables were categorical and were effects coded.

**Table 1:** Attributes and levels for the choice scenarios

Attribute	Description^a^	Levels	Rationale
CAUSE	What is known about the cause of the condition	Unknown Partially known Known	Reflects fear of illnesses that are not well understood and appear to occur at random. Levels are extremes (known or unknown), with an intermediate central level
DIAGNOSIS	How quickly the condition can be diagnosed	Delayed Slightly delayed Rapid	Reflects concern with delayed diagnosis or misdiagnosis, resulting in a delay in patients receiving the right treatment. Levels are extremes (rapid or delayed), with an intermediate central level
PROGNOSIS	Prognosis with current treatments	Death within 2 years Life-long Could recur Curable	Reflects fear of death and also the lifelong heath impact (whether active disease or fear of recurrence). The 2-year duration reflects the life expectancy component of NICE’s end-of-life criteria
CARE	Extent of a patient’s reliance on care as a result of the condition or its treatment	Reliant Sometimes reliant Not reliant	Reflects concerns with loss of independence and dignity, and the effect of caring on family and friends. Middle level describes intense care needed at specific times, such as during treatment, or at end of life
OPTIONS	How the new treatment would fit into the current treatment pathway	Only treatment Further option Additional choice	Reflects need for hope, represented as the length of the treatment pathway. No current treatment (first level) represents little hope, with hope increasing with added lines of treatment (level 2) and availability of alternatives (level 3).
HEALTH	How much a person’s health will improve as a result of the new treatment^b^	0.5 1 5 10	Allows exploration of departure from health maximisation. Continuous variable for use in marginal rate of substitution estimates. Levels chosen to provide a wide range of realistic values

*NICE* National Institute for Health and Care Excellence

^a^Explanations of each attribute and the levels were provided in the survey; the full survey text is available in the electronic supplementary material.

^b^Explained as quality-adjusted life-years in the survey text, but not named as such

### Construction of Choice Tasks

Each choice task was constructed with two alternatives; this is a commonly used structure in healthcare DCEs and aims to keep respondent burden at an acceptable level. Each task was a forced choice with no opt-out; it has been noted that a ‘neither of these’ opt-out is inappropriate if it reflects withholding both treatments when funding is available [28].

### Choice Question

**Fig. 1:**
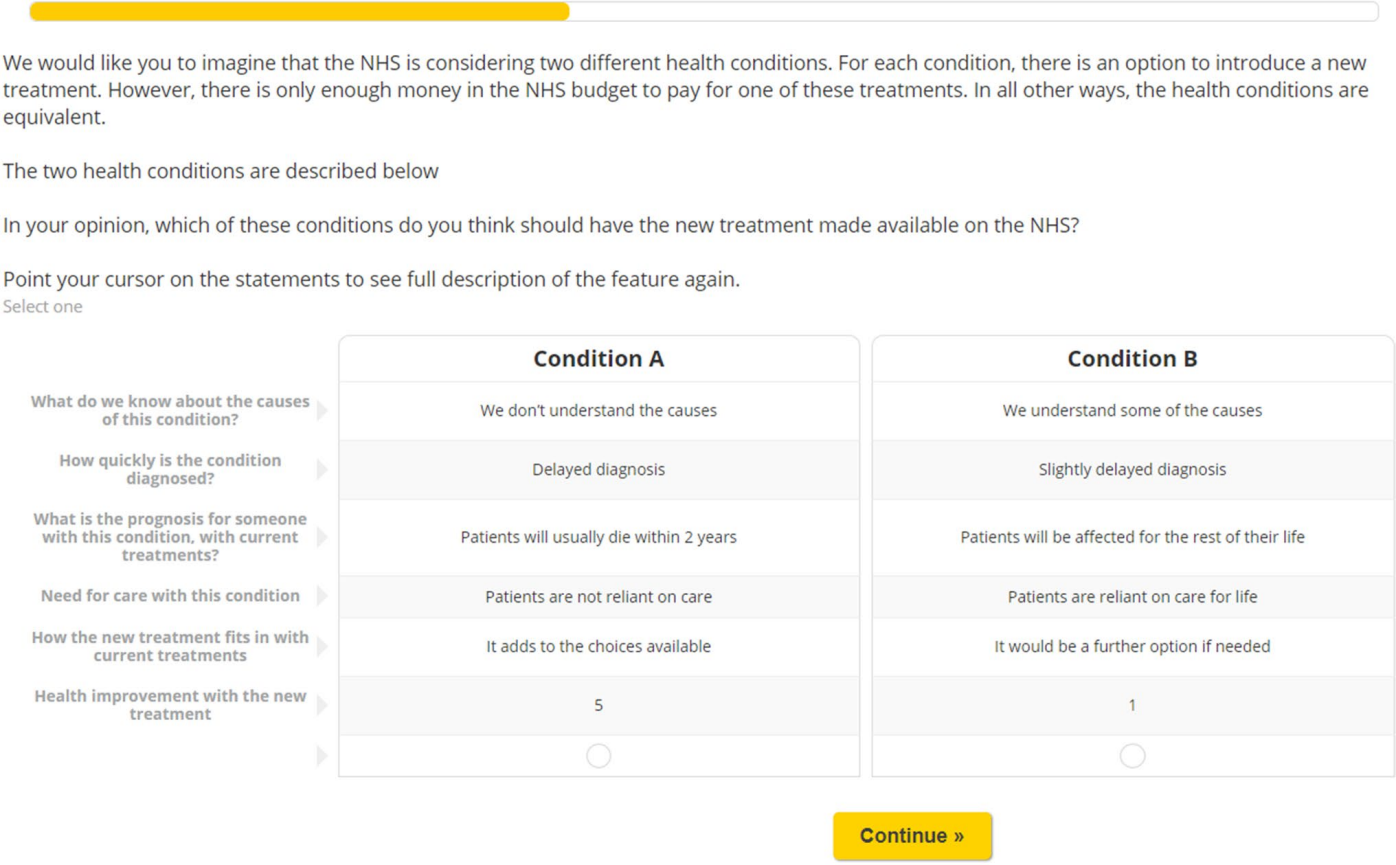
Example choice question shown to respondents. *NHS* National Health Service

Respondents were asked to imagine that the UK National Health Service (NHS) is considering two different health conditions. For each condition, there is an option to introduce a new treatment, but there is only enough funding for one of these treatments. Respondents were presented with profiles for the two conditions side-by-side and asked which condition should have the new treatment made available. An example is shown in Fig. 1. Respondents were asked to respond as themselves, rather than a patient of the condition described, i.e. a socially inclusive personal, ex ante perspective [33].

### Experimental Design

We presented respondents with 15 choice tasks. This number of tasks was chosen to maximise the amount of information generated without excessive burden on respondents, while also optimising the balance of representation of the levels. All respondents saw the same 15 choice tasks.

We produced an efficient DCE design using experimental design software (Ngene [34]). An efficient design generates a set of choice tasks to maximise the amount of information derived from respondents’ choices (specifically, by minimising the D-error, a measure of the standard error of the coefficient estimates). Constraints were applied to avoid implausible scenarios (see the ESM). The most efficient design generated was chosen.

### Survey

The study was presented online and consisted of three sections following an information page and informed consent. Section 1 introduced the need for decisions on which interventions will be funded by the NHS, described the choice that respondents would be asked to make, and provided a description of the attributes and levels. Section 2 provided a practice question, which we constructed to be a straightforward choice, given our hypothesis that respondents would place higher value on more distressing conditions (choice between conditions with the worst levels of each attribute plus large health gain, and the best level of each attribute plus small health gain). The practice question was followed by 15 choice questions. Section 3 contained questions on sociodemographics, current respondent health (EQ-5D-3L), and experience of specific health conditions (see ESM for the survey).

The survey was piloted in a convenience sample (*n* = 14) of adult members of the public contacted through colleagues, friends or family of the project team. Such convenience samples have been used in other healthcare DCEs [22–24, 26]. Minor wording changes were made based on the feedback, and a ‘hover’ function was introduced within the choice tasks, providing additional information to avoid respondents needing to reconsult previous pages.

The study sample size was estimated based on the standard errors predicted from the experimental design [35]. This indicated that a sample size of 900 would allow us to detect a coefficient of absolute value of 0.04, at a significance level of 0.05 and a statistical power of 80%.

### Data Collection

The survey was fielded during November 2017 via an online panel, through ResearchNow, an online market research provider. Panel members were recruited by email and were sampled to be representative of the UK population in terms of sex, age, and country of residence within the UK, based on Office for National Statistics 2016 mid-year population estimates [36].

### Analysis

Data analysis was performed in Stata (v.14SE) [37]. Choice data were initially modelled using a conditional logistic regression model (command ‘clogit’ in Stata), which assumes that all respondents share a common set of preferences. We explored heterogeneity among respondents by relaxing the assumption of common preferences in a random parameters model, which allows for individual-specific variations in preferences (‘mixlogit’). The model estimates both a mean effect and a standard deviation of that effect across respondents, and was estimated using 500 Halton draws. We present this mixed logit as our main analysis.

We further examined heterogeneity with an exploratory latent class analysis to identify subgroups of respondents with similar preferences (‘lclogit’). Models with two to seven classes were estimated and compared using measures of goodness-of-fit—the Akaike, Bayesian, and Consistent Akaike Information Criteria (AIC, BIC and CAIC); lower values of these three measures indicate improved model fit. The preferred model, with the optimal number of classes, was selected as the one that minimised the CAIC.

Although our intention was to model the HEALTH attribute as a continuous linear variable, initial analyses indicated that HEALTH did not have a strictly linear effect on choice probabilities. We therefore present the choice model with HEALTH as a categorical variable; this has little effect on the conclusions but does lead to slightly wider confidence intervals. Preliminary analyses also indicated that the alternative-specific constant was not significantly different from zero (*p* = 0.17). This was expected as the alternatives in the choice questions are not labelled. The alternative-specific constant is therefore excluded from all analyses.

Willingness to forego population health gain to prioritise conditions with a given level relative to the ‘best’ level was estimated by the marginal rate of substitution (MRS) for a health improvement in the range of 1–5 QALYs. This is the difference between the coefficients for a given level and the best level, divided by the rate of change of the HEALTH coefficient between HEALTH=1 and HEALTH=5, i.e. Eq. (1),

1 $$\frac{{\beta }_{\mathrm{worse}}- {\beta }_{\mathrm{best}}}{\left(\frac{{\beta }_{\mathrm{HEALTH}=5}- {\beta }_{\mathrm{HEALTH}=1}}{4}\right)},$$

or using the OPTIONS attribute as an example, 0.33 − (− 0.24) divided by [0.38 − (− 0.06)]/4. This ratio indicates the amount of health that respondents were willing to forgo in order to prioritise a condition with this worse level, holding everything else the same. A negative ratio is interpreted as the amount of additional health that would need to be gained in order to prioritise the condition with the worse level. This is analogous to willingness-to-pay using a cost attribute, but here respondents are effectively ‘paying’ in health opportunity cost. 95% confidence intervals for the MRS were estimated in Stata using the delta method.

To test the robustness of the model, it was re-estimated without respondents who always chose the alternative on the same side of the screen, completed the choice questions in under 1 min (our estimate of a minimum reasonable time to read and respond to 15 questions), or found the survey difficult to complete (responded ‘difficult’ or ‘very difficult’ to the self-reported difficulty question). The model was also estimated without the last 15 respondents aged between 18 and 24 years, as this group was slightly oversampled at recruitment.

Ethical approval was granted by the University of Oxford Medical Sciences Interdivisional Research Ethics Committee (R52559/RE003).

## Results

905 respondents completed the survey, with all respondents completing every question. Compared with the UK general population, the sample was representative for sex and country of residence, but had a slightly higher proportion of respondents under 25 years of age, in higher socioeconomic groups, and educated to degree level (Table 2). Respondents’ self-reported health (both their overall EQ-5D score and their score on the visual analogue scale [VAS]) was lower than the general population. A minority (13.7%) reported finding the survey difficult or very difficult.

**Table 2:** Respondent characteristics

	Respondents [*n* = 905] (%)	UK (%)	Source
*Sex*			
Male	49	49	ONS 2016 [36]
*Age, years*			
18–24	14.4^a^	11.3	ONS 2016 [36]
25–34	17.1	17.2	
35–44	17.1	16.1	
45–54	16.8	17.9	
55–64	13.9	14.7	
65+	20.7	22.8	
*Socioeconomic group*			
ABC1^b^	60.1^a^	55	NRS 2016 [38]
*Education*			
Graduate	37.1^a^	27	Census 2011 [39]
*Country of residence*			
England	83.9	84.2	ONS 2016 [36]
Scotland	8.5	8.2	
Wales	4.8	4.7	
Northern Ireland	2.9	2.8	
*Questionnaire difficulty*			
Very difficult^c^	3.8	–	

	Mean score (SD)	Mean score (SD)	
*EQ-5D*			
Tariff	0.78^a^ (0.28)	0.86 (0.23)	MVH study [40]
VAS	73.9^a^ (20)	82.5 (17)	

*ONS* Office for National Statistics, *NRS* National Readership Survey, *MVH* Measuring and Valuing Health Study, *VAS* visual analogue scale, *SD* standard deviation

^a^Statistically significant (*p* < 0.05) compared with the UK population (comparison of proportions test)

^b^Upper three of six socioeconomic groupings based on occupation, including the person’s qualifications and the number of people they are responsible for

^c^Lowest point on a 7-point scale (very easy to very difficult)

The median time to complete the 15 choice questions was 4 min, with 95% of respondents completing them within 15 min. Ten respondents always chose the alternative on the same side of the screen; we observed that these respondents all took less than 3 min to complete the choice questions, therefore may not have fully considered the questions; however, as we cannot exclude the possibility that these choices reflect genuine preferences, we chose to retain all respondents in the analysis.

### Choice Modelling

#### Logistic regression model (mixed logit)

Table 3 presents the results for the mixed logit regression model. All attributes had a significant effect on preferences. Higher levels of HEALTH had the expected positive sign, showing that the alternative with the higher health gain was more likely to be chosen (had greater utility). For the OPTIONS attribute, the level ‘only treatment’ had a positive sign, indicating that respondents preferred to treat the condition with an unmet need; the other levels had negative coefficients, indicating that conditions that already had treatments available were less likely to be preferred. However, for all other attributes, the best level had a positive sign; respondents were more likely to choose the alternative with the well-understood disease cause, rapid diagnosis, curable prognosis, or where patients were not reliant on care. Alternatives with the worst levels (poorly understood cause, delayed diagnosis, limited life expectancy or lifelong condition, and reliance on care) were not preferred.

**Table 3:** Conditional logistic regression model and willingness to trade health gain

Attribute	Level	Coefficient (95% CI)	Standard deviation (95% CI)^a^	MRS (95% CI)^b^
CAUSE	Unknown	− 0.07 (− 0.13 to − 0.01)	0.24 (0.18 to 0.31)	− 1.5 (− 3.0 to 0.0)
	Partially known	− 0.02 (− 0.07 to 0.02)	0.10 (− 0.02 to 0.22)	− 1.1 (− 2.1 to − 0.1)
	Known^c^	0.09 (0.03 to 0.16)		
DIAGNOSIS	Delayed	0.00 (− 0.14 to 0.14)	− 0.23 (− 0.32 to − 0.14)	− 0.7 (− 2.1 to 0.7)
	Slightly delayed	− 0.07 (− 0.19 to 0.05)	− 0.03 (− 0.11 to 0.04)	− 1.3 (− 3.0 to 0.4)
	Rapid^c^	0.07 (0.01 to 0.13)		
PROGNOSIS	Two years	− 0.85 (− 1.01 to − 0.69)	1.24 (1.14 to 1.34)	− 15.6 (− 25.9 to − 5.4)
	Lifelong	− 0.14 (− 0.25 to − 0.03)	0.34 (0.26 to 0.43)	− 9.2 (− 15.7 to − 2.6)
	Recurrent	0.12 (0.03 to 0.20)	− 0.04 (− 0.18 to 0.09)	− 6.9 (− 11.3 to − 2.5)
	Curable^c^	0.87 (0.68 to 1.06)		
CARE	Reliant	− 0.27 (− 0.38 to − 0.15)	0.36 (0.30 to 0.42)	− 4.0 (− 7.5 to − 0.6)
	Sometimes reliant	0.09 (0.01 to 0.18)	0.01 (− 0.08 to 0.10)	− 0.7 (− 1.8 to 0.3)
	Not reliant^c^	0.17 (0.10 to 0.25)		
OPTIONS	Only treatment	0.33 (0.20 to 0.46)	0.18 (0.09 to 0.28)	5.2 (0.7 to 9.7)
	Further option	− 0.09 (− 0.14 to − 0.04)	0.01 (− 0.08 to 0.09)	1.4 (0.0 to 2.8)
	Additional choice^c^	− 0.24 (− 0.35 to − 0.14)		
HEALTH	0.5	− 0.70 (− 0.94 to − 0.46)	− 0.37 (− 0.46 to − 0.28)	
	1	− 0.06 (− 0.39 to 0.27)	1.08 (0.98 to 1.18)	
	5	0.38 (0.18 to 0.57)	− 0.03 (− 0.23 to 0.17)	
	10^c^	0.39 (0.11 to 0.66)		

CAUSE—what is known about the cause of the condition

DIAGNOSIS—how quickly the condition can be diagnosed

PROGNOSIS—prognosis with current treatments

CARE—the extent of the patient’s reliance on care as a result of the condition or its treatment

OPTIONS—how the new treatment would fit into the current treatment pathway

HEALTH—how much a person’s health will improve as a result of the new treatment

*CI* confidence interval, *MRS* marginal rate of substitution, *QALYs* quality-adjusted life-years

^a^The standard deviation of the mean effect across the population, providing an indication of the heterogeneity of responses

^b^MRS: the number of QALYs that respondents would be prepared to trade in order to choose a condition with the specified level, compared with the best level for that attribute. Positive values indicate the health that respondents would give up to prioritise that level over the best level; negative values indicate the health that respondents would give up to prioritise the best level (or equivalently, the additional health they would need, to choose that level)

^c^Effects-coded attribute. The coefficient for the indicated level is calculated as the negative sum of the coefficients for the other levels of the attribute

For the DIAGNOSIS attribute, the values of the coefficients do not increase consistently when moving from worst to best level; however, the coefficients for ‘delayed’ and ‘slightly delayed’ diagnoses are not significantly different (*p* = 0.61).

Marginal rates of substitution indicate the number of QALYs that respondents would trade to choose their preferred condition. For example, respondents would give up 5.2 QALYs to choose a treatment for an illness with unmet need over one where there was already a choice of treatment options. In contrast, for PROGNOSIS, respondents would choose to treat an end-of-life condition over a curable condition only if it offered over 15 additional QALYs.

The model was robust to the prespecified sensitivity checks and explained 10% of the variation in responses.

#### Latent class model

**Fig. 2:**
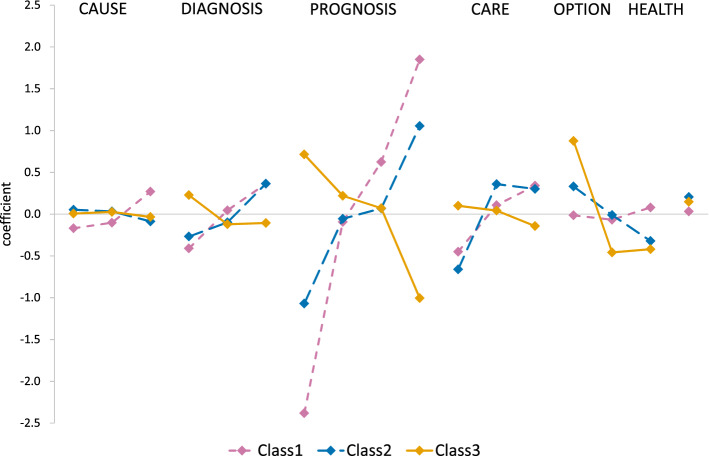
Results of the latent class analysis for three classes of the five-class solution. For each attribute (names at the top of the figure), the regression coefficients for each class are plotted with the levels of all attributes ordered from worst to best as in the preceding tables. The points for each class are joined by a line to show the effect on choices; an upward (downward) sloping line indicates that respondents in this class were more likely to choose to treat a condition that had the better (worse) level of this attribute. Classes 4 and 5 are omitted for clarity.

The model that minimised the CAIC was a five-class solution (Fig. 2) [see the ESM for goodness-of-fit details]. The five-class model included two classes of respondents (Classes 1 and 2 in Fig. 2) who made similar choices to the sample overall but with stronger preferences. In contrast, the Class 3 preferences moved in the opposite direction, with positive coefficients for the worse level across all attributes, i.e. they chose the conditions with poorly understood causes, delayed diagnosis, limited life expectancy, where patients were reliant on care, or there is unmet need.

The remaining classes were either indifferent to the attributes presented (Class 4) or exhibited erratic preferences that were difficult to interpret (Class 5). These are omitted from Fig. 2 for clarity. Details are provided in the ESM.

## Discussion

This study aimed to determine the relative importance of attributes of illness experiences that influence the value placed on alleviation of that illness, in the context of funding prioritisation. Our results show that for most of the attributes studied, members of the public preferred to provide treatments for conditions with the better characteristics: a known cause, rapid diagnosis, curable, and where patients are not reliant on care. In contrast, when considering the available treatment options, respondents preferred to provide treatments for conditions where there is no current treatment, i.e. unmet need, and were prepared to depart from health maximisation (accept lower overall health gain) to do so. A latent class analysis identified a subgroup of respondents who preferred to provide treatments for patients with the worse condition.

In focusing our DCE attributes on the public’s experience and perceptions of distressing health conditions, we provide an alternative perspective on the characteristics of illness that might attract a premium in health technology appraisal. Our work adds to the existing literature considering characteristics of conditions, patients and interventions where there might be a justification to depart from the maxim that QALY = QALY. A similar study in the UK by Rowen et al. [27] used a DCE to evaluate some of the options for weighting factors for the Value-Based Pricing initiative [14]. This study found a preference for weighting health gain at the end of life, and inconsistent results for healthy life-years lost due to illness.

The priority given to better conditions is perhaps surprising, given the responses to the more challenging conditions seen in our qualitative work [30]; respondents talked in emotive terms about the ‘terror’ of a cancer diagnosis or the loss of dignity and independence through dementia or stroke, the ‘lottery’ of incidence of illness, and the shock of an unexpected or delayed diagnosis. Similar emotional power is seen in advocacy for such conditions, and in patient and public response to funding decisions [31]. Typically in priority-setting studies that include aspects of severity of illness, respondents prioritise the more severely ill patient [32]; however, there are examples where respondents do not consistently prioritise patients with lowest quality of life or shortest life expectancy [19, 22, 23, 25].

In terms of technology evaluation, our results do not support a premium for health gain in patients with short life expectancy. This is perhaps not surprising, given the mixed results found in other studies, as reviewed by Shah et al. [41]. Three more recent studies also found no evidence for an end-of-life premium [42–44]. A fourth study did not examine the end-of-life premium in itself but compared preferences for gains through quality of life or life extension [45]; along with two of these more recent papers [43, 44], the authors suggest that gains through improvements in quality of life are preferred to life extension, providing a direct challenge to NICE’s current end-of-life criteria [7].

We do however find that the public place high value on providing treatment for conditions with unmet medical need. This is arguably consistent with studies identifying a preference for inclusive sharing that avoids leaving some groups of patients without treatment [18, 20, 46, 47]. Unmet need is currently handled in UK HTA through the deliberative process. In Scotland, the SMC specifies ‘modifiers’ that can allow acceptance of technologies with a higher cost per QALY than is usually accepted; unmet need is one of those [48]. In England, a NICE appraisal can consider the innovative nature of a technology and any benefits due to innovation that have not been adequately captured elsewhere in the appraisal [7].

The strength of preference to fund a new treatment for a condition that is curable with current treatment, and of the aversion to choosing an end-of-life treatment, is perhaps unexpected. In particular, the estimate of 15 additional QALYs required for respondents to fund an end-of-life condition would be challenging to deliver in a condition with limited life expectancy. It may be that respondents understood this attribute as describing the benefit of the new treatment. However, we did not observe such a misunderstanding during piloting and it was clearly stated in each choice question that the prognosis was with current treatments. This result may indicate the strength of preference for a cure, rather than treatments that simply delay progression or manage symptoms, and we might expect such breakthroughs to be valued by the public beyond their benefits in QALY terms.

Although our respondents as a whole did not prefer to fund treatments for patients in the worst situations, we identified a subgroup of respondents who did. This heterogeneity is not a new finding; subgroups have been found in other DCEs examining prioritisation factors [23, 49], and other methods have been used specifically to identify different viewpoints on health care priorities [50–52]. Shah et al. comment that such heterogeneity may account for the mixed results found in studies evaluating the value that society places on health gain in the last months of life [41]. Heterogeneity clearly creates challenges for policy, with an average or majority-based decision failing to represent all sections of society [53, 54]. Bimodal opposing views, as seen in this study, are particularly challenging. Quantitative studies must be supplemented with other approaches designed to understand the nuances of the alternative positions, and how strongly these views are held, to help translate these findings into a fair and representative policy.

The findings of this study do not align with current policy, in common with much of the body of evidence on public preferences for weighting in technology appraisal. Characteristics currently given formal priority in the UK are cancer [55, 56], end-of-life [7], rarity [12, 57] and large health gain in rare conditions [57]. The empirical evidence finds little support for prioritising these characteristics [20, 27, 32, 41, 58, 59] but does generally support prioritising of severity (although without agreement on how severity is defined) [32, 58, 60] and unmet need [20, 46]. This mismatch between empirical evidence and the public and policy response should be examined further.

### Limitations

A limitation of this study is the high level of unexplained variation in responses, as shown by the low pseudo-*R*^2^ value. DCEs often produce low pseudo-*R*^2^ values, as very strong predictors are required to increase the value of this statistic, and a value of 0.2–0.4 can be considered a good fit [61]. However, our value is low relative to this benchmark, limiting the strength of conclusions we can draw. The exploratory latent class model suggests that part of the variability is due to heterogeneity in respondent views.

Although the study was sampled to be representative of the UK in terms of sex, age and country of residence within the UK, our online sample was younger, slightly better educated, and more likely to be in a higher socioeconomic group, than the population as a whole. This issue is common in online surveys (for example [20, 26, 27]), reflecting access to, or confidence in using, the internet. Our findings may therefore not fully reflect the preferences of older citizens, or of those on lower incomes or with less education, and each of these groups are likely to have specific health concerns [62]. The work could be extended by focusing on these groups, using alternative methods of recruitment and survey delivery, with initial qualitative work to ensure the study is comprehensible and reflects these respondents’ experience. Furthermore, our sample’s self-reported health was lower than the UK average, as defined by the 1993 Measuring and Valuing Health (MVH) study [40]. Rowen et al. reported a similar observation in a health-weighting DCE [27]. It is possible that our sample has poorer health, making their responses less generalisable to the population as a whole. However, this is not what we would expect to see for a younger, better-educated sample, unless our study was of particular interest to a subgroup of the online survey audience who experience poor health. Alternatively, as the MVH study is now over 25 years old, population health on average may have changed and a revised benchmark is needed.

The study design did not allow for interaction between the attributes (for example, between prognosis and the need for care; being reliant on care may be more acceptable over a short life expectancy than for an extended period). To enable exploration of interactions, future studies could use a blocked design, where more choice questions are generated and each respondent is shown only a subset. Furthermore, the order of presentation of choice questions was not randomised, which could have resulted in learning effects that are not accounted for in our analysis.

The practice question was designed to be a relatively straightforward choice, given our initial hypothesis. As respondents did not show all the hypothesised preferences, the practice question may have been more difficult than intended, which may have added to respondent burden or deterred some respondents from completing the study.

## Conclusion

This study suggests a preference among the UK public for treating unmet need; however, it does not support an overall preference for prioritising other distressing aspects of ill health, including no support for giving additional weight to health gain in conditions with limited life expectancy, or where patients are reliant on care. We therefore conclude that all health gains are not considered equal, but perhaps not in the way we might have expected. These results differ from the features currently prioritised in UK health technology appraisal, and the heterogeneity we identify presents a major challenge for the development of broadly accepted policy.

## Supplementary Information

Below is the link to the electronic supplementary material.

Supplementary file1 (DOCX 56 kb)

## Declarations

### Availability of Data and Material

The dataset for this study is available from the corresponding author on reasonable request.
