# Supplementary material for: What Aspects of Illness Influence Public Preferences for Healthcare Priority Setting? A Discrete Choice Experiment in the UK
Source: Pharmacoeconomics. 2021 Aug 19;39(12):1443–54. doi: 10.1007/s40273-021-01067-w (PMC8599241; doi:10.1007/s40273-021-01067-w)
Supplement: Supplementary file 1 — Supplementary file1 (DOCX 56 kb) [file 40273_2021_1067_MOESM1_ESM.docx]

**What aspects of illness influence public preferences for healthcare priority setting? A discrete choice experiment in the UK**

Journal: PharmacoEconomics

Liz Morrell*, James Buchanan, Sian Rees, Richard W. Barker, Sarah Wordsworth

* Health Economics Research Centre, Nuffield Department of Population Health, University of Oxford, Oxford, UK

liz.morrell@ndph.ox.ac.uk

SUPPLEMENTARY MATERIAL

1. DEFINITION OF ATTRIBUTES

2. SURVEY INSTRUMENT

3. EXPERIMENTAL DESIGN: Constraints to avoid implausible combinations

4. MODEL FIT STATISTICS

5. INTERPRETATION OF THE 5-CLASS LATENT CLASS MODEL

1. DEFINITION OF ATTRIBUTES

Three main sources were used to identify attributes of illness that create an emotional response in members of the public, and could affect the value placed on alleviating that illness:

1. A qualitative study of the perceptions of serious illnesses by the public [1]. This identified five themes, shown in the table.
2. Aspects of illness believed to be important, but not fully reflected in technology appraisals. These are derived from a review of the output from the Scottish Medicines Consortium’s ‘Patient and Clinician Engagement’ (PACE) meetings. This forum aims to elicit aspects of value perceived by patients and clinicians that they believe are not fully captured in cost-effectiveness analyses of drugs that the SMC’s New Drugs Committee is minded not to recommend [2]. The table on the following page includes themes related to the characteristics of illnesses that were commonly raised at these meetings. These views from Scotland are assumed to be representative for the UK.
3. A systematic review of public views on weighting factors for healthcare priority setting [3]. The systematic review includes studies from all countries, including the UK; factors from studies other than the UK are included here for breadth. Factors that particularly relate to characteristics of illnesses are included on the following page.

The boxes in the table indicate themes from the various sources – for example, the theme of ‘fear’ from the qualitative work includes fear of death, fear of aggressive and disfiguring treatment, and fear of loss of independence and dignity. The boxes are arranged to line up common or overlapping ideas from the sources. The shading indicates the elements that contributed to the chosen attributes, as described on the following page.

Table S1 Attributes of illness

| **Qualitative work: perceptions of serious illnesses [1]** | **PACE review: factors believed to be important but not reflected in HTA [2]** | **Systematic review of potential weighting factors [3]** | **Additional comments** |
| --- | --- | --- | --- |
| Fear, of:  -death | Short life expectancy, poor prognosis, value of extra time | Short life expectancy | UK HTA agencies have special arrangements for assessing drugs to treat patients at the end of life |
| -symptoms | Severity of symptoms | Severity | No explicit special arrangements in UK HTA, but is in other countries |
| -aggressive treatment | Tolerability, issues with current treatments |  |  |
| -danger to self or others |  |  |  |
| -loss of independence  -loss of dignity | Normality, Independence, Productivity |  | ‘Community and relationships’ not captured in EQ-5D [4] |
| Family as carers |  | Need for care |  |
| Effect on family of illness and death | Effect on family | (Having dependents) |  |
| Hope | Limited options  Little recent development. | Availability of alternative treatment |  |
|  | Hope and reduced anxiety |  |  |
| Recovery | Potential to recover  (in progressive disease) | Cure | ‘Absence of illness’ not captured in EQ-5D [4] |
| Detection |  |  | Themes of late diagnosis, indiscriminate nature, and stealth are elements of ‘cancer fear’ [5] |
| Prevention and Risk | Responsibility | Lifestyle/self-induced illness | risk preconceptions and unpredictability are elements of ‘cancer fear’ [5] |
|  |  | Rarity | UK HTA agencies have special arrangements for assessing drugs to treat rare conditions |

**Attribute development**

Cause of illness (orange shading): this attribute reflects the emotional response to a disease that appears to strike ‘out of the blue’, indiscriminately, with no indication that the person was particularly at risk, or any expectation that their behaviour had contributed.

Diagnosis (yellow shading): this attribute reflects concerns with late diagnosis, by which time treatment opportunities may have been missed. These concerns are common and emotive in discussions of conditions such as cancer or dementia, although less common in discussions of (for example) heart disease.

Prognosis (green shading): this attribute aims to capture a range of prognoses from short life expectancy through chronic or recurring disease to curable. The choice of 2 years as the short life expectancy reflects NICE’s current definition of an ‘end of life’ treatment for consideration by appraisal committees.

Need for care (blue shading): this attribute combines concerns about loss of dignity, independence, normality and productivity, with the impact on family and friends in taking on the role of a carer. The qualitative work highlighted the difference between caring for a patient intensively during specific periods (for example, during chemotherapy treatment, or post-surgery) and long-term care (for example, dementia or post-stroke), in public perceptions of the impact for both patient and carer.

Treatment options (grey shading): this attribute combines the emotional response to unmet need, with few treatment options available or little progress in developing treatments, with the importance of hope and the ability to take action in supporting mental well-being during illness. The attribute reflects ‘hope’ as the length of the treatment pathway; no current treatments reflects little hope, [1]with hope increasing with added lines of treatment, and with having a choice of options at a given stage on the pathway.

Health gain: this attribute is included in the DCE reflecting the maximand of current HTA processes, which aim to maximise health from a defined health budget. The study will explore the extent to which respondents are willing to trade off health gain, in order to alleviate particular aspects of illness.

Concern with ‘aggressive’ or poorly tolerated treatments was common to both the qualitative work and the PACE meetings. This was challenging to operationalise as an attribute in the DCE, without introducing the idea of a new treatment, and the aim of the study was to focus on characteristics of the illness rather than new treatments. For this reason, this attribute was not included in the study.

In addition to the role of family in providing care, all sources made some reference to the broader impact of illness on family, and on dependents. These included (for example) describing life a ‘on hold’ or ‘devastated’ during a relative’s illness, increased concerns for their own risk levels, and concerns for the care for children. Without negating these issues, their construction in an attribute would be on based on the individual circumstances, rather than characteristic of the illness. Having captured part of the impact on family in the attribute relating to care, we chose not to include further attributes on family impact.

Rare diseases receive particular attention in HTA policy, with adaptations to the appraisal process in the UK, and weighting factors for rarity have been considered in multiple studies [3]. However it did not appear as a factor in the qualitative work, or in the PACE meeting review. The issues of rare diseases, however, are captured within the other attributes: for rare childhood metabolic diseases or cancers, for example, there may be a random mutation causing the condition, a long diagnostic odyssey, limited life expectancy, few treatment options, and need for lifelong care. As these features are already present, rarity itself was not added as an attribute.

The qualitative work discussed conditions where a patient is a risk to themselves or to others – for example, mental illness or dementia. As this was only mentioned in one of the sources used, this was not included in the DCE.

SOURCES

1. Morrell L, Ii SS, Wordsworth S, Wilson R, Rees S, Barker R. Cancer as the “perfect storm”? A qualitative study of public attitudes to health conditions. Health Science Reports. 2017. doi:10.1002/hsr2.16.

2. Morrell L, Wordsworth S, Fu H, Rees S, Barker R. Cancer drug funding decisions in Scotland: impact of new end-of-life, orphan and ultra-orphan processes. BMC Health Services Research. 2017;17(1):613. doi:10.1186/s12913-017-2561-0.

3. Gu Y, Lancsar E, Ghijben P, Butler JRG, Donaldson C. Attributes and weights in health care priority setting: A systematic review of what counts and to what extent. Social Science & Medicine. 2015;146 41-52. doi:10.1016/j.socscimed.2015.10.005.

4. Vrinten C, McGregor LM, Heinrich M, von Wagner C, Waller J, Wardle J et al. What do people fear about cancer? A systematic review and meta-synthesis of cancer fears in the general population. Psycho-Oncology. 2016. doi:10.1002/pon.4287.

5. Shah KK, Mulhern B, Longworth L, Janssen MF. Views of the UK General Public on Important Aspects of Health Not Captured by EQ-5D. Patient. 2017;10(6):701-9. doi:10.1007/s40271-017-0240-1.

2. SURVEY INSTRUMENT

**WELCOME PAGE**

Welcome to this survey on the National Health Service (NHS) in the United Kingdom.

In a publicly funded health service like the NHS, decisions have to be made about which treatments should be made available to patients. This survey aims to understand which illnesses and treatments members of the public would prefer to see funded.

You have been invited to participate as a member of the public, and a potential user of the NHS. We will show you a series of descriptions of different health conditions, in pairs, and ask which of the pair you would prefer the NHS to treat. The descriptions are all fictional – they are not about ‘real’ individual patients in the NHS. The results will help to inform NHS policies for the future.

This survey is being run by the researchers at the Radcliffe Department of Medicine, University of Oxford. We are interested in your views about how money should be spent in the NHS. Please note that your participation is entirely voluntary.

Please try to answer all of the questions in this survey. There are no ‘correct’ answers – we are just interested in your views. To help you to answer the questions we have provided some background information at the start of the survey. Please read this carefully before you complete the survey.

This survey should take around 20 minutes to complete. You may withdraw at any point during the survey for any reason, before submitting your answers, by closing your browser window.

The principal researcher is Dr. Liz Morrell. Colleagues at the University may assist with the statistical analysis of the data. The survey responses will be collected by [supplier] through the [panel name]. Your answers will be completely anonymous, and we will use all reasonable endeavours to keep them confidential. Responses to the survey will be collected into a single data file which does not identify any individual, transferred to the University researcher, and stored for 5 years as a password-protected file. Your IP address will not be stored by the University. The data will only be used for research purposes, and the results may be used in academic publications. Your information will not be shared with any other institutions.

This project has been reviewed by, and received ethics clearance through, the University of Oxford Central University Research Ethics Committee [R52559/RE003].

**What if there is a problem?**

Please contact [supplier] on [helpdesk email]. If they cannot resolve your concern, they will contact the lead researcher and work with her to deal with your concern.. If you remain unhappy or wish to make a formal complaint, [supplier] will provide you with contact details for the Chair of the Research Ethics Committee at the University of Oxford

Having read the information on the previous page, do you agree to participate in this study?

☐ Yes, I agree to take part

**THANK YOU FOR YOUR HELP**

**ABOUT THIS SURVEY**

In this survey we would like you to think about the National Health Service (NHS) in the United Kingdom.

In a publicly funded health service like the NHS, decisions have to be made about which treatments should be made available to patients. This survey aims to understand which illnesses and treatments members of the public would prefer to see funded.

We would like you to imagine that the NHS is considering two different health conditions. For each condition, there is an option of introducing a new treatment, but there is only enough money in the NHS budget to pay for one of these treatments. We will show you a side-by-side description of the two conditions and potential treatments, and we would like you to choose which treatment you would prefer to be made available to NHS patients. We would like you to answer based on your views, as a member of the public, and a potential user of the NHS, now or in the future.

The health conditions are described by 6 different features. In all other respects, the two health conditions in each scenario are equivalent. The descriptions and choices are all fictional – they do not relate to specific patients or treatments in the NHS today.

The following pages provide more information about the choices that we will ask you to make, and the health conditions that we will describe to you.

**THANK YOU FOR YOUR HELP**

[Note for scripting: features described in the next section, to be displayed one per screen]

The first feature is how much we know about WHAT CAUSES THE ILLNESS. For each health condition there are three options:

UNKNOWN We do not understand what causes this condition. People might describe this as ‘it seems to strike out of the blue’

PARTIALLY KNOWN We understand some of the causes of this condition. People might describe this as ‘it seems to be partly down to luck’

KNOWN We fully understand the causes of this condition

The second feature relates to the DIAGNOSIS of the condition – how quickly a doctor will be able to tell a person exactly what their condition is. Timely diagnosis is important because it means that a patient will receive the correct treatment as soon as possible. For each health condition there are three options:

DELAYED DIAGNOSIS Patients eventually receive the correct diagnosis, but by this time the patient will have been ill for a long time. There will have been a delay in starting the right treatment, and the patient will have tried many other treatments that did not work for them.

SLIGHTLY DELAYED DIAGNOSIS Patients receive the correct diagnosis, but by this time the patient will have been ill for a short time. There will have been a short delay in starting the right treatment, and the patient will have tried a small number of other treatments that did not work for them.

RAPID DIAGNOSIS Patients receive the correct diagnosis straight away, and the correct treatment can start immediately without trying other treatments first.

The third feature is PROGNOSIS – what is likely to happen to a person with the condition, with the current treatments. For each health condition there are four options:

DEATH WITHIN 2 YEARS A person with this condition will usually die within 2 years of diagnosis, with the current treatments.

LIFE-LONG A person with this condition cannot be cured with current treatments, and will be affected by symptoms for the rest of their life.

COULD RECUR A person with this condition will recover after receiving the current treatment. However, the condition could come back again later in life.

CURABLE A person with this condition will be completely cured after receiving the current treatment

The fourth feature is NEED FOR CARE – the extent to which a person with this condition will be reliant on others for care and support as a result of having this condition, or of having treatment for it. Needing care can have an effect on the person who is ill, and also on their family and friends. For each health condition there are three options:

RELIANT A person with this condition will be reliant on care and support from relatives or other carers for the rest of their life.

SOMETIMES RELIANT A person with this condition will be reliant on care and support from relatives or other carers at certain times during their illness, for example, when undergoing and recovering from treatment.

NOT RELIANT A person with this condition is completely independent and not reliant on relatives or others for care

For the fifth feature, remember we asked you to imagine that the NHS was considering introducing a new treatment for one of these health conditions. This feature is about HOW THE NEW TREATMENT WOULD FIT IN with any treatments already available for this condition. For each health condition there are three options:

ONLY TREATMENT The new treatment would be the only treatment available for this condition.

FURTHER OPTION The new treatment would add an option for future treatment, if the disease were to get worse or come back again.

ADDITIONAL CHOICE The new treatment would add to the choices available to treat this condition now.

The final feature describes HOW MUCH A PERSON’S HEALTH WILL IMPROVE as a result of the new treatment, compared with current treatments. We will use a health improvement score that takes account of both how long someone lives, and how well they are during their life. As a result of a treatment, a person could either live longer, or feel better, or both. Each of those kinds of health improvement will contribute to the health improvement score. We don’t need more details of the calculations for this study: the important things to know are:

0.5

- A higher score means more health improvement for the patient
- The scores work like normal numbers, so 10 is twice as much health improvement as 5

For each health condition, the health improvement score for a patient on the new treatment is one of four options:

0.25

0.5 A small health improvement, such as living for an extra year in moderate health, or improving from poor to good health for 1 year

1 A modest health improvement, such as living for an extra 2 years in moderate health, or improving from poor to good health for 2 years

5 A good health improvement, such as living for an extra 10 years in moderate health, or improving from poor to good health for 10 years.

10 A major health improvement, such as living for an extra 20 years in moderate health, or improving from poor to good health for 20 years.

Some general points:

1. You will notice that we don’t name the health conditions. For this study it doesn’t really matter what the condition is called – we are interested in which treatment you would prefer to be funded based on the description we have given.

2. We also don’t specify what the new treatment is. When we say ‘treatment’, that could mean any kind of medical intervention – so it could be drugs, surgery, prevention, talking therapy: whatever is appropriate for that particular condition. Again, the nature of the treatment doesn’t matter for our study.

3. In the choice questions we will ask you, there is no ‘I can’t decide’ option. We would like you to make a choice for each question. That’s because in this situation in real life, a decision would have to be made. There is definitely enough money in the NHS budget to provide one of the treatments, so the NHS cannot fail to decide, and end up providing neither treatment. In the same way, we would like you to make a choice for each question, even if you find the decision difficult.

**PRACTICE QUESTION**

Before we start on the main study, we would like you to complete a practice choice question

We would like you to imagine that the NHS is considering two different health conditions. For each condition, there is an option to introduce a new treatment. However, there is only enough money in the NHS budget to pay for one of these treatments. In all other ways, the health conditions are equivalent.

The two health conditions are described below

|  | CONDITION A | CONDITION B |
| --- | --- | --- |
| What do we know about the causes of this condition? | We don’t understand the causes | We fully understand the causes |
| How quickly is the condition diagnosed? | Delayed diagnosis | Rapid diagnosis |
| What is the prognosis for someone with this condition, with current treatments? | Patients will usually die within 2 years | Patients will be cured |
| Need for care with this condition | Patients are reliant on care for life | Patients are reliant on care at certain times during their illness |
| How the new treatment fits in with current treatments | It would be the only treatment | It adds to the choices available |
| Health improvement with the new treatment | 10 | 1 |

In your opinion, which of these conditions do you think should have the new treatment made available on the NHS?

| Condition A |  | Condition B |  |
| --- | --- | --- | --- |

**MAIN QUESTIONS**

Thank you for completing the practice question.

Now we would like you to complete the main part of the survey.

We are going to present you with 15 scenarios that are similar to the practice question.

Each scenario asks you to make the same choice about which treatment should be made available to NHS patients. However, the exact features of the health conditions will be different each time.

Even if you find it difficult to decide between the two health conditions presented, or would not make a new treatment available in either case, please indicate which you would prefer if you had to choose one.

**QUESTION 1**

We would like you to imagine that the NHS is considering two different health conditions. For each condition, there is an option to introduce a new treatment. However, there is only enough money in the NHS budget to pay for one of these treatments. In all other ways, the health conditions are equivalent.

The two health conditions are described below

|  | CONDITION A | CONDITION B |
| --- | --- | --- |
| What do we know about the causes of this condition? | We understand some of the causes | We fully understand the causes |
| How quickly is the condition diagnosed? | Rapid diagnosis | Slightly delayed diagnosis |
| What is the prognosis for someone with this condition, with current treatments? | Patients will be cured | Patients will usually die within 2 years |
| Need for care with this condition | Patients are reliant on care at certain times during their illness | Patients are reliant on care for life |
| How the new treatment fits in with current treatments | It adds to the choices available | It would be the only treatment |
| Health improvement with the new treatment | 5 | 1 |

In your opinion, which of these conditions do you think should have the new treatment made available on the NHS?

| Condition A |  | Condition B |  |
| --- | --- | --- | --- |

(etc to QUESTION 15)

**RESPONDENT CHARACTERISTICS**

Now we would like to ask some questions about you.

All of the information that you provide will help us in our analysis, and all of your details will remain confidential.

If you do not wish to answer some of these questions you do not have to.

How old are you? (standard categories)

What is your gender? (standard categories)

Socioeconomic group (standard categories)

Educational level (standard categories)

Location (England/Scotland/Wales/Northern Ireland)

Health state (EQ5D, slider response for VAS)

Experience of specific health conditions in the past 12 months – personally, in immediate family, as a carer (grid layout)

cancer, heart disease, stroke, mental illness, dementia

How easy or difficult did you find the questions in this survey?

[Likert scale 1-7, 1 = very easy, 7 = very difficult]

Are there any further comments that you would like to make regarding this survey? (free text)

You have reached the end of the survey. Thank you for taking the time to participate.

3. EXPERIMENTAL DESIGN: Constraints to avoid implausible combinations

1. If the level for Prognosis was ‘Curable’ then the following four attribute levels were not options:

- Diagnosis: No test (We decided it didn’t make sense to have a curable condition for which there is no diagnostic. It would also add to respondent burden if we had to explain the alternative e.g. diagnosis by elimination);
- Need for care: A person with this condition will be reliant on care and support from relatives or other carers for the rest of their life (No care required if cured, but patient could require care during/post treatment, e.g. surgery, so occasional reliance on care could still be reasonable);
- Role of the new treatment: The new treatment would be the only treatment available for this condition (Clearly not an option if the condition is currently curable);
- Role of the new treatment: The new treatment would add an option for further treatment, if the disease were to get worse or come back again later in life (This is clearly not an option if the condition is currently curable).

2. If the level for Prognosis was ‘Could recur’ then the following three attribute levels were not options:

- Diagnosis: No test (We decided it didn’t make sense to have a recurrent condition for which there is no diagnostic. It would also add to respondent burden if we had to explain the alternative e.g. diagnosis by elimination);
- Need for care: A person with this condition will be reliant on care and support from relatives or other carers for the rest of their life (Not possible, as they will live some of their life not suffering from symptoms);
- Role of the new treatment: The new treatment would be the only treatment available for this condition (Not an option if the symptoms of the condition can be resolved currently, even if they recur).

4. MODEL FIT STATISTICS

Table S2 Measures of model fit

| Model |  | AIC | BIC | CAIC | pseudo-R^2^ |
| --- | --- | --- | --- | --- | --- |
| Conditional logit* (with ASC) | | 17384 | 17507 |  | 0.0779 |
| Conditional logit* (no ASC) | | 17391 | 17506 |  | 0.0774 |
| Mixed logit* (no ASC) | | 15759 | 15989 |  | 0.0956 |
| Latent class: | 2-class | 16199 | 16199 | 16394 |  |
|  | 3-class | 15561 | 15561 | 15858 |  |
|  | 4-class | 15561 | 15561 | 15783 |  |
|  | 5-class | 15210 | 15210 | 15709 |  |
|  | 6-class | 15150 | 15150 | 15752 |  |

* HEALTH attribute as a categorical variable

AIC – Akaike Information Criterion

BIC - Bayesian Information Criterion

CAIC - Constant Akaike Information Criterion

Lower values of these measures indicate improved model fit

ASC – alternative-specific constant

The mixed logit, no-ASC model, with the HEALTH attribute included as a categorical variable, is the main analysis presented in the paper.

5. INTERPRETATION OF THE 5-CLASS LATENT CLASS MODEL

The 5-class model identifies 2 classes of respondents (Classes 1 and 2) who make similar choices to the sample overall, but with stronger preferences; that is they show a preference for the ‘better off’ condition for attributes CAUSE, DIAGNOSIS, PROGNOSIS, and CARE, and for higher levels of HEALTH gain. Class 1 shows a more extreme preference than Class 2 for PROGNOSIS. These classes differ in their responses on the OPTION attribute; Class 2 appear indifferent to level of unmet need, with Class 4 showing a strong preference to treat the condition where there is no other treatment. Class 2 also shows a stronger preference for increased HEALTH gain, resulting in lower marginal rates of substitution for health; Class 2 are willing to give up less health in order to prioritise their preferred level on another attribute, than Class 1 (Table S1).

In contrast, the preferences of Class 3 moved in the opposite direction, with positive coefficients for the ‘worse case’ condition across all attributes; that is, Class 3 chose the conditions with poorly understood causes, delayed diagnosis, limited life expectancy, or where patients were reliant on care, in addition to unmet need. Notably, treating an end-of-life condition is worth an additional 11.6 QALYs for Class 3. Class 3 is one of the smallest groups accounting for 13% of the sample. (Table S1)

The remaining classes were either indifferent to the attributes presented (Class 4, accounting for nearly a third of the sample), or exhibited erratic preferences that were difficult to interpret (Class 5, 11%).

Table S3 Latent class analysis – five-class model

|  |  | Coefficients | | | | | MRS | | | | |
| --- | --- | --- | --- | --- | --- | --- | --- | --- | --- | --- | --- |
| Attribute | Level | Class1 | Class2 | Class3 | Class4 | Class5 | Class1 | Class2 | Class3 | Class4 | Class5 |
| CAUSE | unknown | -0.168* | 0.053 | 0.006 | -0.077** | -0.336 | -14 | 0.7 | 0.3 | 182 | 0.2 |
|  | partially known | -0.103 | 0.033 | 0.027 | 0.047 | 0.765* | -12 | 0.6 | 0.4 | -29 | 2.1 |
|  | known^a^ | 0.271* | -0.086 | -0.034 | 0.030 | -0.429 | - | - | - | - | - |
| DIAGNOSIS | delayed | -0.410** | -0.267* | 0.227* | -0.009 | 0.5804 | -24 | -3.1 | 2.3 | 39 | 0.7 |
|  | slightly delayed | 0.046 | -0.097 | -0.121 | -0.005 | -0.785* | -10 | -2.3 | -0.1 | 32 | -1.7 |
|  | rapid^a^ | 0.365* | 0.365* | -0.106* | 0.014 | 0.204 | - | - | - | - | - |
| PROGNOSIS | 2 years | -2.382** | -1.069** | 0.713* | -0.123* | -0.401 | -131 | -10.4 | 11.6 | 544 | -2.2 |
|  | lifelong | -0.093 | -0.058 | 0.219 | -0.055 | 0.370 | -60 | -5.4 | 8.3 | 429 | -0.8 |
|  | recurrent | 0.624** | 0.071 | 0.072 | -0.018 | -0.818 | -38 | -4.8 | 7.3 | 365 | -2.9 |
|  | curable^a^ | 1.851** | 1.055* | -1.005* | 0.195* | 0.849 | - | - | - | - | - |
| CARE | reliant | -0.450 | -0.661* | 0.100 | -0.059 | -1.596** | -25 | -4.7 | 1.6 | 159 | -2.9 |
|  | sometimes reliant | 0.108 | 0.359* | 0.042 | 0.026 | 1.513** | -7 | 0.3 | 1.3 | 14 | 2.5 |
|  | not reliant^a^ | 0.341 | 0.302 | -0.142 | 0.034 | 0.082 | - | - | - | - | - |
| PATH | only treatment | -0.014 | 0.331* | 0.875* | 0.105* | -0.235 | -3 | 3.2 | 8.8 | -353 | 0.1 |
|  | further option | -0.066 | -0.010 | -0.457* | -0.003 | 0.546 | -5 | 1.5 | -0.3 | -168 | 1.5 |
|  | additional choice^a^ | 0.080 | -0.321* | -0.41 | -0.101 | -0.311 | - | - | - | - | - |
| HEALTH | per QALY | 0.032* | 0.205* | 0.148* | -0.001 | 0.570** | - | - | - | - | - |
|  |  |  |  |  |  |  |  |  |  |  |  |
| Class share |  | 22% | 21% | 13% | 32% | 11% |  |  |  |  |  |

* p-value < 0.05, ** p-value < 0.01

a effects-coded attribute. The coefficient for the indicated level is calculated as the negative sum of the coefficients for the other levels of the attribute

MRS: marginal rate of substitution – the amount of HEALTH that respondents would be prepared to trade in order to choose a condition with the specified level, compared to the ‘best’ level for that attribute. Positive values indicate the health (QALY’s) respondents would give up to prioritise that level over the ‘best’; negative values indicate the health they would give up to prioritise the ‘better’ level (or equivalently, the additional QALY’s they would need, to choose that level).

Class shares are the average probabilities across respondents, of belonging to that class.
